# Geographic and socio-demographic predictors of household food insecurity in Canada, 2011–12

**DOI:** 10.1186/s12889-018-6344-2

**Published:** 2019-01-03

**Authors:** Valerie Tarasuk, Andrée-Anne Fafard St-Germain, Andrew Mitchell

**Affiliations:** Department of Nutritional Sciences, Faculty of Medicine, University of Toronto, 1 King’s College Circle, Toronto, ON M5S 1A8 Canada

**Keywords:** Household food insecurity, Income, Social assistance, Indigenous peoples, Canada

## Abstract

**Background:**

Household food insecurity is a potent social determinant of health and health care costs in Canada, but understanding of the social and economic conditions that underlie households’ vulnerability to food insecurity is limited.

**Methods:**

Data from the 2011–12 Canadian Community Health Survey were used to determine predictors of household food insecurity among a nationally-representative sample of 120,909 households. Household food insecurity over the past 12 months was assessed using the 18-item Household Food Security Survey Module. Households were classified as food secure or marginally, moderately, or severely food insecure based on the number of affirmative responses. Multivariable binary and multinomial logistic regression analyses were used to determine geographic and socio-demographic predictors of presence and severity of household food insecurity.

**Results:**

The prevalence of household food insecurity ranged from 11.8% in Ontario to 41.0% in Nunavut. After adjusting for socio-demographic factors, households’ odds of food insecurity were lower in Quebec and higher in the Maritimes, territories, and Alberta, compared to Ontario. The adjusted odds of food insecurity were also higher among households reliant on social assistance, Employment Insurance or workers’ compensation, those without a university degree, those with children under 18, unattached individuals, renters, and those with an Aboriginal respondent. Higher income, immigration, and reliance on seniors’ income sources were protective against food insecurity. Living in Nunavut and relying on social assistance were the strongest predictors of severe food insecurity, but severity was also associated with income, education, household composition, Aboriginal status, immigration status, and place of residence. The relation between income and food insecurity status was graded, with every $1000 increase in income associated with 2% lower odds of marginal food insecurity, 4% lower odds of moderate food insecurity, and 5% lower odds of severe food insecurity.

**Conclusions:**

The probability of household food insecurity in Canada and the severity of the experience depends on a household’s province or territory of residence, income, main source of income, housing tenure, education, Aboriginal status, and household structure. Our findings highlight the intersection of household food insecurity with public policy decisions in Canada and the disproportionate burden of food insecurity among Indigenous peoples.

**Electronic supplementary material:**

The online version of this article (10.1186/s12889-018-6344-2) contains supplementary material, which is available to authorized users.

## Background

Household food insecurity, the inadequate or insecure food access due to financial constraints, is increasingly recognized as a serious population health problem in affluent countries. Awareness has typically been driven by reports of increased demands for charitable food assistance [1–9]. In Canada, demands for charitable food assistance began to escalate in the 1980s [10], and population-level assessment of food insecurity commenced in the 1990s [11], with systematic measurement introduced in 2004 [12]. The most recent national data suggest that 12.4% of Canadian households were food insecure in 2011–12 [13], with a fourfold difference in prevalence across the individual provinces and territories that comprise Canada [14]. Food insecurity is most prevalent in Canada’s most northern territory, Nunavut, where the population is predominantly Inuit and faces high living costs and high risk of poverty [15]. While household food insecurity clearly reflects material deprivation, the problem is important in its own right because food insecurity has adverse effects on health that are independent of other measures of low socioeconomic status. In Canada, household food insecurity is associated with heightened nutritional vulnerability [16], increased risk of numerous physical and mental health problems [17–25], higher mortality rates [26], and higher health care costs [16, 27], independent of income, education, and other social determinants of health. Consistent with evidence from the U.S. [28–32], the relationship between food insecurity and health in Canada is graded, with more severe food insecurity associated with greater likelihood of negative health outcomes [20, 22, 25, 26] and higher health care costs [16].

Despite more than two decades of population-level measurement, the conditions that give rise to household food insecurity remain poorly understood and food insecurity reduction has not been a priority for public policy intervention [33, 34]. Although some provincial policy decisions have been associated with changes in food insecurity rates [35–37], there has been little analysis of how vulnerability relates to province or territory of residence. Multivariable analyses of earlier national surveys documented associations between various indicators of food insecurity and low income, social assistance, Aboriginal status, renting rather than owning one’s dwelling, and lone-parent female-led families [17, 21, 38], but these studies offer limited insight into the determinants of food insecurity or directions for intervention. The household circumstances, contextual factors, and public policies that impact food insecurity prevalence in the U.S. have been studied extensively (e.g., [39–45]), but extrapolations from this research to other countries are limited by interjurisdictional differences in public policy.

Understanding the household characteristics associated with increased risk of food insecurity and more severe food insecurity in Canada and the role of province or territory of residence in relation to this problem is prerequisite to identifying the determinants of this population health problem. Drawing on data from the most recent nationally-representative population survey to assess food insecurity, we describe the social and geographic patterning of household food insecurity and severity of food insecurity in Canada.

## Methods

The 2011–12 Canadian Community Health Survey (CCHS) was a population-representative survey of individuals 12 years and older, excluding individuals who were full-time members of the Canadian Forces, lived on First Nations Reserves, Crown Lands or in some remote regions of Quebec, or were in prisons or care facilities [46]. Altogether, these exclusions represent less than 3% of the population [46]. Household food security status over the past 12 months was assessed using the Household Food Security Survey Module (HFSSM) (Additional file 1), a well-validated, 18-item scale of severity developed by the U.S. Department of Agriculture (USDA) to monitor food insecurity in that country [47–49]. Our study was limited to households with complete data on the HFSSM (approximately 96% of the entire sample), *n* = 120,909.

Households with any affirmative response on the HFSSM were considered food insecure, recognizing the growing body of evidence indicating that even a single affirmative response on the HFSSM denotes significant vulnerability [50, 51], with important consequences for health and well-being [16, 22, 52, 53]. Moderate and severe household food insecurity were determined using the classification scheme developed by Health Canada [12]. Households with one affirmative response were classified as marginally food insecure. It should be noted that the thresholds and terminology applied to define household food insecurity status based on this scale are normative [54], and Health Canada’s classification scheme differs from USDA’s [40]. In Canada, the adult and child sub-scales of the module are considered separately in determining household food insecurity status, whereas in the US food insecurity status is a function of the number of affirmatives on the entire module. Additionally, in Canada, the terms ‘moderate’ and ‘severe food insecurity’ are used to differentiate levels of severity of food insecurity, whereas in the US, severity is framed in terms of ‘low’ or ‘very low food security’. (The Canadian and US classification schemes are summarized in Additional file 2.)

Because food insecurity is assessed at the household level, we focused on variables measured at this level: before-tax household income, main source of income, household structure, highest level of education in the household, home ownership, urban versus rural residence, and province or territory of residence. Household income was adjusted for household size by dividing by the square root of the number of persons in the household [55], the standard method to account for economies of scale to monitor low-income prevalence in Canada [56]. In addition, we included the Aboriginal identity and immigration status of the respondent as proxies for the household. For all categorical variables, the category with the largest number of observations served as the reference group. Approximately 30% of the sample did not report income, and Statistics Canada imputed values for this group. For all other variables, missing responses were coded as such to minimize sample loss and preserve the information provided by every observation.

Logistic regression models were run first individually for each socio-demographic characteristic to generate unadjusted odds ratios of food insecurity. A multivariable logistic regression was then run including all of the variables considered to yield adjusted odds ratios of food insecurity.

To identify geographic and socio-demographic characteristics associated with severity of food insecurity, a multinomial logistic regression model was run, regressing the four-level variable for household food insecurity status (i.e., food secure, marginally food insecure, moderately food insecure, and severely food insecure) on the afore-mentioned geographic and socio-demographic characteristics.

All analyses were conducted in SAS version 9.4, using SURVEY commands with bootstrap replication (*n* = 500) and bootstrap weights, provided by Statistics Canada. Institutional ethics approval for this study was received from the Human Research Ethics Board of the University of Toronto.

## Results

**Fig. 1:**
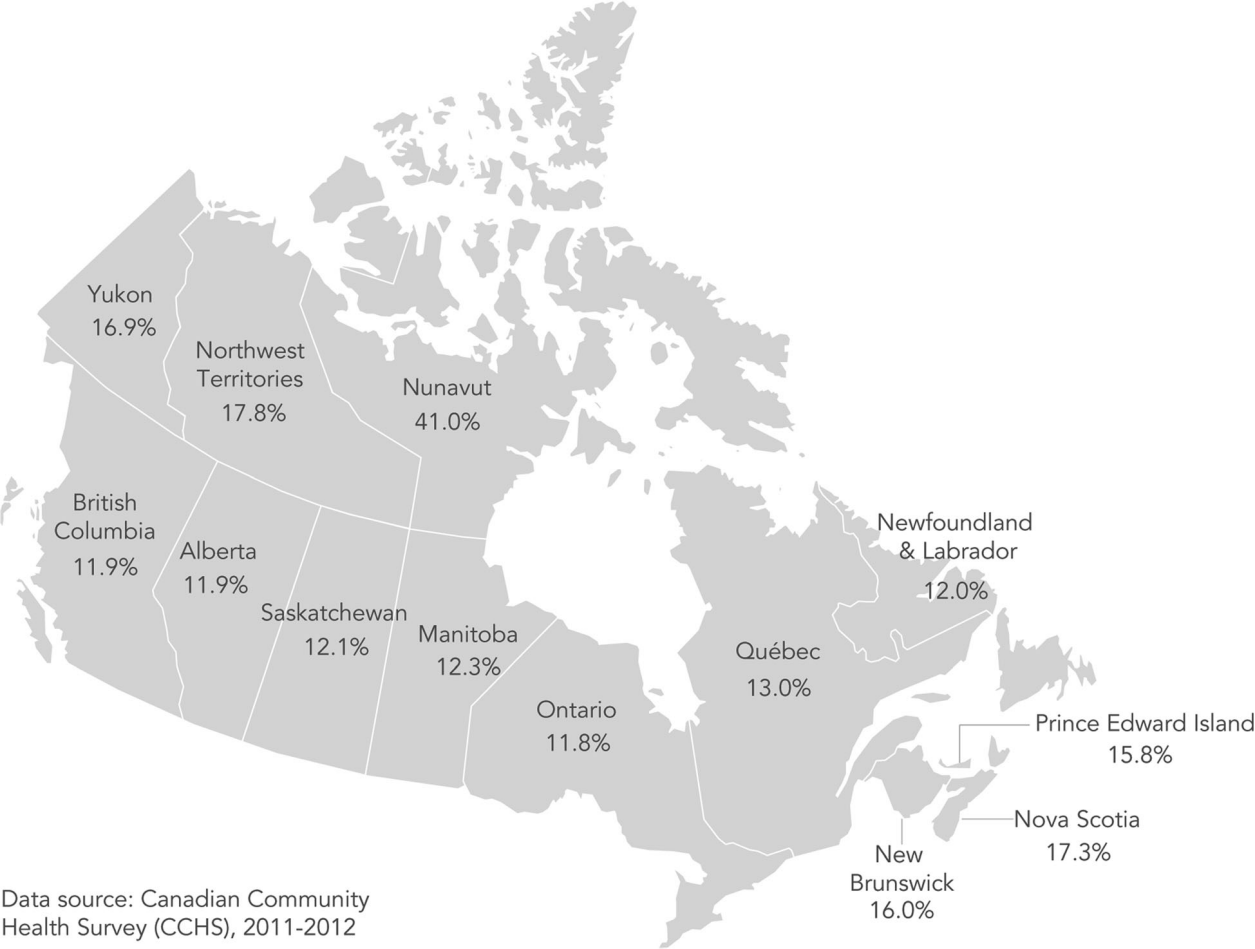
Prevalence of household food insecurity by province and territory, 2011–12

The prevalence of household food insecurity ranged from 11.8% in Ontario to 41.0% in Nunavut (Fig. 1). Table 1 presents the distribution of household food insecurity status by province/territory of residence and household socio-demographic characteristics.

**Table 1:** Socio-demographic characteristics of households, by household food insecurity status, Canada, 2011–12 (*n* = 120,909)

	Food secure	Food insecure		
		Marginal	Moderate	Severe
Weighted N (000 s)	11,468.6	536.2	759.6	333.5
Provinces and territories, %				
Newfoundland and Labrador	88.0%	4.4%	5.9%	1.8%
Prince Edward Island	84.2%	5.2%	7.8%	2.7%
Nova Scotia	82.7%	5.4%	8.1%	3.8%
New Brunswick	84.0%	5.9%	7.1%	3.1%
Quebec	87.0%	4.9%	5.8%	2.3%
Ontario	88.2%	3.6%	5.6%	2.7%
Manitoba	87.7%	4.3%	6.1%	1.8%
Saskatchewan	87.9%	4.0%	5.8%	2.4%
Alberta	88.1%	3.8%	5.9%	2.3%
British Columbia	88.1%	3.6%	5.3%	2.9%
Yukon	83.1%	5.5%	8.5%	3.0%
Northwest Territories	82.2%	4.1%	9.4%	4.3%
Nunavut	59.1%	4.3%	19.0%	17.7%
Household income^a^, mean ± SEM	$52,975 ± 438	$30,275 ± 580	$24,778 ± 570	$18,888 ± 454
Main source of household income, %				
Wages/salaries or self-employment	89.1%	4.1%	5.2%	1.6%
Seniors’ income, including pensions, dividends and interest	92.8%	2.7%	3.2%	1.4%
Employment insurance or workers’ compensation	62.4%	10.0%	17.3%	10.3%
Social assistance	32.9%	8.3%	30.4%	28.4%
Other or none^b^	70.7%	9.7%	12.7%	7.0%
Missing	91.1%	3.5%	4.5%	0.9%
Education (highest level in household), %				
Less than completed high school	79.1%	4.6%	10.6%	5.7%
Completed high school	83.6%	4.8%	7.9%	3.7%
Some post-secondary	76.8%	6.4%	10.0%	6.9%
Completed post-secondary, below bachelor’s degree	86.7%	4.5%	6.2%	2.6%
Bachelor’s degree or higher	93.9%	2.8%	2.6%	0.7%
Missing	87.0%	4.8%	6.2%	2.0%
Household structure^c^, %				
Unattached, living alone or with others	83.4%	4.7%	7.2%	4.8%
Couple, no children	94.4%	2.2%	2.7%	0.8%
Couple with children	89.6%	4.1%	5.2%	1.1%
Female lone parent	72.3%	8.1%	13.3%	6.3%
Male lone parent	84.8%	5.1%	8.3%	1.9%
Other or missing	84.8%	5.3%	8.1%	1.8%
Housing tenure, %				
Owns dwelling	93.5%	2.8%	2.9%	0.9%
Renter	74.4%	7.0%	12.3%	6.3%
Missing	84.8%	6.7%	5.2%	3.4%
Aboriginal status of respondent, %				
Non-aboriginal	88.1%	4.0%	5.5%	2.3%
Aboriginal	72.3%	5.4%	14.1%	8.1%
Immigration status of respondent, %				
Canadian born	87.7%	4.0%	5.7%	2.7%
Immigrant < 10 years	81.9%	7.4%	8.4%	2.3%
Immigrant, ≥ 10 years	89.6%	3.4%	5.1%	1.9%
Missing	81.0%	5.1%	9.7%	4.3%
Urban/rural residence, %				
Population centre	87.2%	4.1%	6.0%	2.8%
Rural	89.4%	4.1%	4.9%	1.6%

^a^Before-tax income, adjusted for family size by dividing by the square root of household size

^b^‘Other or none’ includes child benefits, child support and alimony

^c^Households identified as including children were those with at least one person under the age of 18

## Presence of food insecurity

Table 2 presents crude and adjusted odds ratios of household food insecurity for the variables considered. Adjusting for household characteristics had a marked effect on the odds ratios of food insecurity in two jurisdictions: the odds ratio associated with living in Nunavut fell from 5.20 (95% CI: 4.08, 6.64) to 2.85 (95% CI: 2.02, 4.02), and the odds ratio associated with living in Quebec fell from 1.12 (95% CI: 1.03, 1.22) to 0.89 (95% CI: 0.81, 0.97). Households in the Maritimes (i.e., Nova Scotia, New Brunswick and Prince Edward Island), Alberta, Northwest Territories, and Yukon also had significantly higher adjusted odds of food insecurity than households in Ontario.

**Table 2:** Crude and adjusted odds of food insecurity in relation to socio-demographic characteristics, Canada, 2011–12 (*n* = 120,909)

	OR (95% CI)	
	Crude	Adjusted
Provinces and territories		
Newfoundland and Labrador	1.02 (0.87–1.20)	0.86 (0.74–1.04)
Prince Edward Island	1.41 (1.16–1.71)	1.34 (1.10–1.63)
Nova Scotia	1.57 (1.37–1.79)	1.50 (1.30–1.74)
New Brunswick	1.43 (1.27–1.61)	1.38 (1.22–1.56)
Quebec	1.12 (1.03–1.22)	0.89 (0.81–0.97)
Ontario	1.00	1.00
Manitoba	1.05 (0.90–1.22)	0.99 (0.85–1.16)
Saskatchewan	1.03 (0.88–1.21)	1.00 (0.86–1.15)
Alberta	1.01 (0.89–1.15)	1.34 (1.16–1.53)
British Columbia	1.01 (0.91–1.12)	1.03 (0.92–1.15)
Yukon	1.53 (1.27–1.85)	1.27 (1.03–1.57)
Northwest Territories	1.62 (1.28–2.05)	1.54 (1.24–1.92)
Nunavut	5.20 (4.08–6.64)	2.85 (2.02–4.02)
Household income^a^	0.95 (0.95–0.96)	0.97 (0.96–0.97)
Main source of household income		
Wages/salaries or self-employment	1.00	1.00
Seniors’ income, including pensions, dividends and interest	0.63 (0.58–0.69)	0.41 (0.37–0.46)
Employment insurance or workers compensation	4.93 (3.99–6.08)	2.14 (1.70–2.70)
Social assistance	16.69 (14.73–18.91)	3.24 (2.79–3.75)
Other or none^b^	3.39 (2.87–4.01)	1.16 (0.96–1.39)
Missing	0.80 (0.69–0.92)	0.47 (0.40–0.55)
Education (highest level in household)		
Less than completed high school	4.06 (3.60–4.57)	1.54 (1.33–1.77)
Completed high school	3.02 (2.69–3.39)	1.48 (1.30–1.70)
Some post-secondary	4.64 (4.00–5.38)	1.86 (1.57–2.21)
Completed post-secondary, below bachelors degree	2.36 (2.13–2.60)	1.61 (1.45–1.80)
Bachelor’s degree or higher	1.00	1.00
Missing	2.30 (1.98, 2.66)	1.48 (1.26, 1.75)
Household structure ^c^		
Unattached, living alone or with others	3.35 (3.06–3.66)	1.58 (1.43–1.75)
Couple, no children	1.00	1.00
Couple with children	1.94 (1.77–2.14)	1.56 (1.40–1.73)
Female lone parent	6.41 (5.70–7.22)	1.98 (1.74–2.26)
Male lone parent	3.02 (2.32–3.93)	1.44 (1.10–1.87)
Other or missing	3.01 (2.34–3.87)	2.03 (1.55–2.66)
Housing tenure		
Owner	1.00	1.00
Renter	4.94 (4.63–5.26)	2.33 (2.16–2.52)
Missing	2.57 (1.71–3.87)	1.46 (0.95–2.24)
Cultural/racial identity		
Non-aboriginal	1.00	1.00
Aboriginal	2.84 (2.55–3.17)	1.54 (1.34–1.78)
Immigrant		
Canadian born	1.00	1.00
Immigrant < 10 years	1.57 (1.35–1.82)	0.91 (0.76–1.08)
Immigrant, ≥ 10 years	0.83 (0.75–0.91)	0.89 (0.80–0.99)
Missing	1.67 (1.15–2.42)	1.19 (0.82–1.72)
Urban/rural residence		
Population centre	1.00	1.00
Rural	0.81 (0.75–0.87)	0.99 (0.91–1.07)

^a^Before-tax income, in thousands of Canadian dollars, adjusted for family size by dividing by the square root of household size

^b^‘Other or none’ includes child benefits, child support and alimony

^c^Households identified as including children were those with at least one person under the age of 18

Our multivariable analysis also revealed significantly higher odds ratios of food insecurity among households reliant on social assistance or Employment Insurance or workers’ compensation, those in which no one had a university degree, those including children under 18 or comprising an unattached individual, those who rented rather than owned their dwelling, and those with an Aboriginal respondent (Table 2). Having a higher income, being reliant on seniors’ income sources, and having immigrated to Canada ten or more years ago were protective against food insecurity (Table 2).

Adjustment for province or territory and the full spate of household characteristics considered in our multivariable model yielded large decreases in the strength of some effects, highlighting the co-occurrence of factors associated with elevated risk. Compared to households reliant on employment incomes, the odds ratio of food insecurity associated with reliance on social assistance fell from 16.69 (95% CI: 14.73, 18.91) to 3.24 (95% CI: 2.79, 3.75) and the odds ratio associated with reliance on Employment Insurance or workers’ compensation fell from 4.93 (95% CI: 3.99, 6.08) to 2.14 (95% CI: 1.70, 2.70) with adjustment. The odds ratio of food insecurity associated with being a female-led lone-parent household also fell from 6.41 (95% CI: 5.70, 7.22) to 1.98 (95% CI: 1.74, 2.26) with adjustment for other household characteristics.

## Severity of food insecurity

In Table 3, the adjusted odds ratios of marginal, moderate, and severe household food insecurity relative to food security are presented for each of the geographic and sociodemographic variables considered here. Living in Nova Scotia or Alberta (versus Ontario) was associated with higher odds ratios of all three levels of food insecurity, but being in Prince Edward Island and New Brunswick was only associated with elevated odds ratios of marginal and moderate food insecurity. Residents of Northwest Territories and Nunavut had significantly elevated odds ratios of moderate and severe food insecurity, with the odds of severe food insecurity in Nunavut 6.16 times (95% CI: 3.39, 11.21) as high as in Ontario. Living in Quebec versus Ontario was associated with 18% lower odds of moderate food insecurity and 41% lower odds of severe food insecurity. Compared to Ontario, the odds of severe food insecurity were also 52 and 40% lower for those in Newfoundland and Labrador and Manitoba, respectively.

**Table 3:** Adjusted odds of marginal, moderate, and severe household food insecurity in relation to household socio-demographic characteristics, Canada, 2011–12

	Marginal food insecurity	Moderate food insecurity	Severe food insecurity
Provinces and territories			
Newfoundland and Labrador	1.11 (0.86–1.44)	0.86 (0.68–1.10)	0.48 (0.30–0.76)
Prince Edward Island	1.40 (1.03–1.89)	1.39 (1.07–1.80)	1.11 (0.75–1.65)
Nova Scotia	1.56 (1.26–1.93)	1.50 (1.22–1.84)	1.41 (1.07–1.86)
New Brunswick	1.66 (1.36–2.01)	1.27 (1.07–1.50)	1.16 (0.89–1.53)
Quebec	1.17 (1.02–1.34)	0.82 (0.72–0.94)	0.59 (0.48–0.73)
Ontario	1.00	1.00	1.00
Manitoba	1.20 (0.95–1.52)	1.01 (0.82–0.25)	0.60 (0.43–0.83)
Saskatchewan	1.15 (0.92–1.46)	0.98 (0.78–1.22)	0.78 (0.59–1.04)
Alberta	1.29 (1.06–1.56)	1.40 (1.18–1.66)	1.32 (1.02–1.69)
British Columbia	1.02 (0.86–1.21)	0.99 (0.85–1.16)	1.12 (0.92–1.37)
Yukon	1.55 (1.14–2.10)	1.28 (0.94–1.74)	0.84 (0.58–1.23)
Northwest Territories	1.33 (0.94–1.89)	1.63 (1.22–2.19)	1.80 (1.12–2.89)
Nunavut	1.46 (0.96–2.22)	2.63 (1.83–3.78)	6.16 (3.39–11.21)
Household income^a^	0.98 (0.97–0.98)	0.96 (0.96–0.97)	0.95 (0.94–0.96)
Main source of household income			
Wages/salaries or self-employment	1.00	1.00	1.00
Seniors’ income, including pensions, dividends and interest	0.50 (0.43–0.58)	0.38 (0.32–0.44)	0.35 (0.28–0.44)
Employment insurance or workers compensation	1.87 (1.28–2.73)	2.01 (1.46–2.79)	2.82 (2.04–3.89)
Social assistance	1.58 (1.25–2.02)	2.79 (2.28–3.43)	5.18 (4.04–6.65)
Other or none^b^	1.28 (0.97–1.68)	1.02 (0.80–1.30)	1.15 (0.82–1.62)
Missing	0.57 (0.45–0.72)	0.48 (0.38–0.61)	0.25 (0.18–0.35)
Education (highest level in household)			
Less than completed high school	1.07 (0.85–1.33)	1.98 (1.61–2.43)	1.83 (1.34–2.49)
Completed high school	1.20 (0.99–1.46)	1.73 (1.43–2.08)	1.83 (1.33–2.52)
Some post-secondary	1.47 (1.14–1.90)	1.97 (1.56–2.49)	2.85 (2.04–3.98)
Completed post-secondary, below bachelors degree	1.32 (1.13–1.54)	1.83 (1.55–2.15)	2.15 (1.67–2.76)
Bachelor’s degree or higher	1.00	1.00	1.00
Missing	1.31 (1.06–1.63)	1.62 (1.26–2.10)	1.79 (1.26–2.57)
Household structure ^c^			
Unattached, living alone or with others	1.52 (1.30–1.77)	1.34 (1.16–1.55)	2.60 (2.10–3.21)
Couple, no children	1.00	1.00	1.00
Couple with children	1.65 (1.40–1.94)	1.59 (1.38–1.84)	1.15 (0.90–1.48)
Female lone parent	2.19 (1.80–2.65)	1.83 (1.53–2.20)	2.08 (1.56–2.75)
Male lone parent	1.58 (1.10–2.25)	1.51 (1.04–2.17)	0.97 (0.52–1.81)
Other or missing	2.05 (1.34–3.16)	2.15 (1.46–3.18)	1.55 (0.76–3.19)
Housing tenure			
Owner	1.00	1.00	1.00
Renter	1.91 (1.70–2.14)	2.69 (2.41–3.01)	2.47 (2.08–2.94)
Missing	1.69 (0.92–3.10)	1.08 (0.58–2.00)	2.04 (0.66–6.30)
Cultural/racial identity			
Non-aboriginal	1.00	1.00	1.00
Aboriginal	1.16 (0.96–1.40)	1.65 (1.38–1.97)	1.87 (1.47–2.38)
Immigrant			
Canadian born	1.00	1.00	1.00
Immigrant < 10 years	1.18 (0.95–1.48)	0.87 (0.70–1.08)	0.55 (0.36–0.83)
Immigrant, ≥ 10 years	0.91 (0.77–1.07)	0.94 (0.81–1.09)	0.74 (0.58–0.95)
Missing	1.15 (0.70–1.91)	1.29 (0.69–2.43)	1.02 (0.42–2.49)
Urban/rural residence			
Population centre	1.00	1.00	1.00
Rural	1.10 (0.99–1.23)	0.98 (0.88–1.00)	0.77 (0.66–0.91)

^a^Before-tax income, in thousands of Canadian dollars, adjusted for family size by dividing by the square root of household size

^b^‘Other or none’ includes child benefits, child support and alimony

^c^Households identified as including children were those with at least one person under the age of 18

A gradient was apparent in the relationship between income and severity of food insecurity, with every one thousand dollars increase in before-tax income associated with 2% lower odds of marginal food insecurity (OR: 0.98; 95% CI: 0.97, 0.98), 4% lower odds of moderate food insecurity (OR: 0.96; 95% CI: 0.96, 0.97), and 5% lower odds of severe food insecurity (OR: 0.95; 95% CI: 0.94, 0.96) (Table 3). Severity was also related to the main source of household income. Compared to households reliant on employment incomes, the odds of severe food insecurity were 5.18 times (95% CI: 4.04, 6.65) as high among social assistance recipients and 65% lower (OR: 0.35; 95% CI: 0.28, 0.44) among households reliant on seniors’ income sources.

Education, household composition, housing tenure, Aboriginal status, immigration status, and rural/urban residence also related differently to different levels of food insecurity (Table 3). Compared to couples without children, all other household types had higher odds of marginal and moderate food insecurity, but only female lone-parent households and unattached individuals had significantly higher odds of severe food insecurity. Being Aboriginal was associated with elevated odds ratios of moderate and severe, but not marginal food insecurity. Compared to Canadian-born respondents, immigrants had significantly lower odds of severe food insecurity, irrespective of whether they had come within the last 10 years or earlier, but immigration status was not associated with marginal or moderate food insecurity. Similarly, living in a rural area was associated with lower odds of severe food insecurity compared to living in an urban area, but unrelated to marginal or moderate food insecurity.

## Discussion

The probability of household food insecurity in Canada and the severity of the experience are tightly linked to the province or territory of residence, as well as to household income, main source of income, housing tenure, education, Aboriginal status, and household structure. Although we examined a broader array of socio-demographic variables than most prior studies, our findings indicate that the risk factors identified in earlier analyses [17, 20, 21, 38, 57–59] remain important indicators of vulnerability.

The importance of household income, home ownership, and main source of income as predictors of household food insecurity status is consistent with research suggesting that households’ vulnerability is a function of their capacity to avoid or weather negative income shocks (e.g., sudden losses in income or increased expenses) [43, 60, 61]. Independent of income, home ownership indicates an asset that affords protection against transitory income shocks, while also insulating owners from the inflationary pressures to which renters are subject [62]. The lower risk of food insecurity among households reliant on seniors’ incomes is consistent with research positing that the public pensions provided to Canadians over 65 years of age are protective against food insecurity because of the income adequacy and security they offer [63, 64]. This protection stands in contrast to the elevated risk of food insecurity among households on social assistance, Employment Insurance, and workers’ compensation – three other public income support programs. Social assistance, a means-tested cash transfer program administered by the provinces and territories, is the program of ‘last resort’ for working-aged adults in Canada, but over two-thirds of recipients report some food insecurity. The five-fold drop in the odds ratios of food insecurity after adjustment for income and other socio-demographic characteristics suggests that a large part of their elevated risk stems from social assistance recipients’ very low benefit levels and greater likelihood of bearing other characteristics associated with food insecurity (e.g., renting rather than owning their dwellings). Observed reductions in food insecurity among social assistance recipients in Newfoundland and Labrador and British Columbia following improvements to benefits [35, 36] further support the interpretation that the very high rate of food insecurity among this group is largely a function of benefit levels. The fact that social assistance recipients still had greater odds ratio of food insecurity after adjustment for income and other household characteristics may indicate unobserved selection effects and/or other defining features of social assistance not captured by the variables available in this survey. For example, we were unable to control for households’ savings or assets (except home ownership), but the stringent asset limits of social assistance programs [65] and the inability of people with such low incomes to ever amass savings mean they are very unlikely to have a financial cushion against which to buffer financial shocks.

Our results add to earlier studies documenting extreme levels of vulnerability among Inuit populations in Nunavut [66–69] by highlighting the marked disparity between Nunavut and the rest of Canada even after interjurisdictional differences in the composition of the population are taken into account. Although our multivariable models included household income, we were unable to identify a means to account for interjurisdictional differences in the costs of basic necessities and thus could not assess the adequacy of household incomes relative to living costs. This is particularly salient given the high food costs in Canada’s North [15]. More research is needed to understand the extent to which the elevated rate of food insecurity in Nunavut relates to the greater prevalence of households with incomes insufficient to meet basic living costs in that jurisdiction.

Other interjurisdictional differences in food insecurity risk observed after taking into account compositional differences in the provinces and territories may in part reflect differences in macroeconomic conditions across jurisdictions, but they likely also relate to interjurisdictional differences in the supports provided to at-risk groups. For example, minimum wages [70] and social assistance programs [65] differ considerably across the provinces and territories. Policy decisions that impact costs of basic necessities also impact food insecurity rates, as indicated by one Canadian study reporting an effect of differences in provincial responses to a sharp spike in heating costs in 2000–01 on food insecurity rates over that period [37]. The apparent protection conferred by living in Quebec, also observed in an earlier study of food insecurity in Canada’s labour force [58], may reflect more generous social programs in that province, but research is needed to confirm this. More research is also needed to determine what accounts for the other provincial and territorial differences charted here and to identify policies and programs that are mitigating risk in some jurisdictions.

The greater likelihood of food insecurity among Aboriginal groups has been documented previously [15, 17, 21, 38, 57, 71], and our results confirm the persistence of this inequity. Importantly, we found significantly higher odds of moderate and severe food insecurity among households with an Aboriginal respondent even after adjusting for household income, province/territory of residence, and several other socio-demographic variables. The elevated risk is concerning because food insecurity is a potent determinant of health and well-being among Aboriginal people [72, 73]. Even when considered in conjunction with measures of familial attendance at residential schools and a myriad of other structural and social determinants of health, food insecurity is strongly associated with lower self-perceived health and poorer mental health among off-reserve First Nations, Metis, and Inuit adults [72]. At a time when the Canadian government is embarking on a new relationship with Indigenous peoples [74], it is critically important that their extreme vulnerability to food insecurity be recognized and addressed.

Marginally food insecure households have traditionally been treated as part of the food secure population [12], but our results indicate that they represent a distinct group. The socio-demographic predictors of marginally food insecure households suggest that they are, on average, less disadvantaged than moderately and severely food insecure households, but have lower incomes than food secure households and are more likely to bear sociodemographic characteristics associated with elevated risk of food insecurity. These results, taken in tandem with findings that marginal food insecurity is associated with poorer health [16, 22, 52, 53] and higher health care utilization [16, 25], argue for an end to the practice of treating marginally food insecure households as if they are food secure.

Strengths of this study include the large, population-representative sample, use of a well-validated scale to assess household food insecurity, inclusion of a broad spectrum of socio-demographic characteristics, and examination of both food insecurity presence and severity. We were limited, however, by the lack of specificity in some key variables. For example, we could not identify refugees, a group whose vulnerability to food insecurity may be distinct from immigrants, nor were we able to differentiate social assistance recipients on welfare from those receiving disability benefits (i.e., programs with very different benefit levels). We also could not account for the health or disability status of household members because we lacked health information for anyone other than the respondent. The presence of members with chronic health problems has been found to independently increase the risk and severity of household food insecurity in Canada [20, 75–77] and the US [78–81]. In addition, our results are limited by the fact that almost one-third of our sample did not report their incomes. We retained households with missing data in order to maximize our sample size, but given prior analyses of Canadian Community Health Survey data indicating that failure to report one’s income is associated with significantly lower odds ratio of food insecurity even after other socio-demographic characteristics have been taken into account [35, 36], it is likely that we have underestimated the true relationship between income and household food insecurity status. Finally, it is important to acknowledge that our analyses are purely descriptive, and inferences are limited by the cross-sectional nature of the data.

## Conclusions

Our study has delineated the complex array of household characteristics that predict household food insecurity in Canada, highlighting the particular disadvantage of residents of Nunavut, households reliant on social assistance and Indigenous populations. Our findings lay the foundation for future work to identify specific directions for intervention. The marked differences in risk associated with households’ reliance on different publicly funded income support programs speak to the link between problems of household food insecurity and public policy decisions. Our finding that households’ risks of food insecurity also depends on which province or territory they inhabit points to the need for more research to understand how policies and practices at this level of government shape household food insecurity prevalence and severity.

## Additional files

Additional file 1: Household Food Security Survey Module. (DOCX 17 kb)

Additional file 2: Determination of household food insecurity status from the Household Food Security Survey Module. (DOCX 14 kb)

## Abbreviations

CCHS: Canadian Community Health Survey
CI: Confidence Interval
HFSSM: Household Food Security Survey Module
USDA: United States Department of Agriculture
