# Supplementary material for: Geographic and socio-demographic predictors of household food insecurity in Canada, 2011–12
Source: BMC Public Health. 2019 Jan 3;19:12. doi: 10.1186/s12889-018-6344-2 (PMC6318847; doi:10.1186/s12889-018-6344-2)
Supplement: Supplementary file 1 — Household Food Security Survey Module. (DOCX 17 kb) [file 12889_2018_6344_MOESM1_ESM.docx]

**Additional file 1:**

**Household Food Security Survey Module [12]**

The following questions are about the food situation for your household in the past 12 months.

**Q1. Which of the following statements best describes the food eaten in your household in the past 12 months, that is since [current month] of last year?**

1. You and other household members always had enough of the kinds of foods you wanted to eat.

2. You and other household members had enough to eat, but not always the kinds of food you wanted.

3. Sometimes you and other household members did not have enough to eat.

4. Often you and other household members didn’t have enough to eat.

- Don’t know / refuse to answer (Go to end of module)

*Question Q1 is not used directly in determining household food security status.*

**STAGE 1 Questions 2 - 6 — ask all households**

Now I’m going to read you several statements that may be used to describe the food situation for a household. Please tell me if the statement was often true, sometimes true, or never true for you and other household members in the past 12 months.

**Q2. The first statement is: you and other household members worried that food would run out before you got money to buy more. Was that often true, sometimes true, or never true in the past 12 months?**

1. Often true 3. Never true

2. Sometimes true - Don’t know/refuse to answer

**Q3. The food that you and other household members bought just didn’t last, and there wasn’t any money to get more. Was that often true, sometimes true, or never true in the past 12 months?**

1. Often true 3. Never true

2. Sometimes true - Don’t know/refuse to answer

**Q4. You and other household members couldn’t afford to eat balanced meals. In the past 12 months was that often true, sometimes true, or never true?**

1. Often true 3. Never true

2. Sometimes true - Don’t know/refuse to answer

--IF CHILDREN UNDER 18 IN HOUSEHOLD, ASK Q5 AND Q6; OTHERWISE, SKIP TO FIRST LEVEL SCREEN—

Now I’m going to read a few statements that may describe the food situation for households with children.

**Q5. You or other adults in your household relied on only a few kinds of low-cost food to feed the child(ren) because you were running out of money to buy food. Was that often true, sometimes true, or never true in the past 12 months?**

1. Often true 3. Never true

2. Sometimes true - Don’t know/refuse to answer

**Q6. You or other adults in your household couldn’t feed the child(ren) a balanced meal, because you couldn’t afford it. Was that often true, sometimes true, or never true in the past 12 months?**

1. Often true 3. Never true

2. Sometimes true - Don’t know/refuse to answer

**FIRST LEVEL SCREEN (screener for Stage 2):** If AFFIRMATIVE RESPONSE to ANY ONE of Q2-Q6 (i.e., “often true” or “sometimes true”) OR response [3] or [4] to Q1, then continue to STAGE 2; otherwise, skip to end.

**STAGE 2 Questions 7 - 11 — ask households passing the First Level Screen**

--IF CHILDREN UNDER 18 IN HOUSEHOLD, ASK Q7; OTHERWISE SKIP TO Q8—

**Q7. The child(ren) were not eating enough because you and other adult members of the household just couldn’t afford enough food. Was that often, sometimes or never true in the past 12 months?**

1. Often true 3. Never true

2. Sometimes true - Don’t know/refuse to answer

The following few questions are about the food situation in the past 12 months for you or any other adults in your household.

**Q8. In the past 12 months, since last [current month] did you or other adults in your household ever cut the size of your meals or skip meals because there wasn’t enough money for food?**

1. Yes

2. No (Go to Q9)

- Don’t know / refuse to answer

**Q8b. How often did this happen?**

1. Almost every month 3. Only 1 or 2 months

2. Some months but not every month - Don’t know/ refuse to answer

**Q9. In the past 12 months, did you (personally) ever eat less than you felt you should because there wasn’t enough money to buy food?**

1. Yes

2. No

- Don’t know / refuse to answer

**Q10. In the past 12 months, were you (personally) ever hungry but didn’t eat because you couldn’t afford enough food?**

1. Yes

2. No

- Don’t know / refuse to answer

**Q11. In the past 12 months, did you (personally) lose weight because you didn’t have enough money for food?**

1. Yes

2. No

- Don’t know / refuse to answer

**SECOND LEVEL SCREEN (screener for Stage 3):** If AFFIRMATIVE RESPONSE to ANY ONE of Q7-Q11, then continue to STAGE 3; otherwise, skip to end.

**STAGE 3 Questions 12 - 16 — ask households passing the Second Level Screen**

**Q12. In the past 12 months, did you or other adults in your household ever not eat for a whole day because there wasn’t enough money for food?**

1. Yes

2. No (IF CHILDREN UNDER 18 IN HOUSEHOLD, ASK Q13; OTHERWISE SKIP TO END)

- Don’t know / refuse to answer

**Q12b. How often did this happen?**

1. Almost every month 3. Only 1 or 2 months

2. Some months but not every month -Don’t know/ refuse to answer

--IF CHILDREN UNDER 18 IN HOUSEHOLD, ASK Q13-16; OTHERWISE SKIP TO END—

Now, a few questions on the food experiences for children in your household.

**Q13. In the past 12 months, did you or other adults in your household ever cut the size of any of the children’s meals because there wasn’t enough money for food?**

1. Yes

2. No

- Don’t know / refuse to answer

**Q14. In the past 12 months, did any of the children ever skip meals because there wasn’t enough money for food?**

1. Yes

2. No

- Don’t know / refuse to answer

**Q14b. How often did this happen?**

1. Almost every month 3. Only 1 or 2 months

2. Some months but not every month - Don’t know/ refuse to answer

**Q15. In the past 12 months, were any of the children ever hungry but you just couldn’t afford more food?**

1. Yes

2. No

- Don’t know / refuse to answer

**Q16. In the past 12 months, did any of the children ever not eat for a whole day because there wasn’t enough money for food?**

1. Yes

2. No

- Don’t know / refuse to answer

**END OF MODULE**

Reference List

(1) Health Canada. Canadian Community Health Survey, Cycle 2.2, Nutrition (2004) - Income-Related Household Food Security in Canada. Ottawa, ON: Office of Nutrition Policy and Promotion,Health Products and Food Branch,Health Canada; 2007. Report No.: 4696.
