# Supplementary material for: Geographic and socio-demographic predictors of household food insecurity in Canada, 2011–12
Source: BMC Public Health. 2019 Jan 3;19:12. doi: 10.1186/s12889-018-6344-2 (PMC6318847; doi:10.1186/s12889-018-6344-2)
Supplement: Supplementary file 2 — Determination of household food insecurity status from the Household Food Security Survey Module. (DOCX 14 kb) [file 12889_2018_6344_MOESM2_ESM.docx]

**Additional File #2:**

**Determination of household food security status, based on household food security survey module**

| Household Status | 18 Item Household Food  Security Scale | | 10 Item Adult Food Security Scale | 8 Item Child Food Security Scale |
| --- | --- | --- | --- | --- |
| Canada^1,2^ | | | | |
| Food secure | - | | No items affirmed on either scale | |
| Marginal food insecurity | - | | No more than 1 item affirmed on  either scale | |
| Moderate food insecurity | - | | 2 to 5 positive responses | 2 to 4 positive responses |
| Severe food insecurity | - | | 6 or more positive responses | 5 or more positive responses |
| United States^3^ | | | | |
| Food secure | No more than 2 items affirmed | | - | - |
| Food insecure,  Low food security | *Households without children* | 3 to 5 positive responses | - | - |
|  | *Households with children* | 3 to 7 positive responses |  |  |
| Food insecure,  Very low food security | *Households without children* | 6 or more positive responses | - | - |
|  | *Households with children* | 8 or more positive responses |  |  |

^1^In rows with more than one condition for classification, meeting *either* condition is sufficient for classification into the category. In cases where a household meets the conditions of two different classifications, the household status is that of the more severe classification.

^2^Adapted from: Canadian Community Health Survey, Cycle 2.2, Nutrition (2004): Income-related Household Food Security in Canada.

^3^Adapted from: Coleman-Jensen A, Rabbitt MP, Gregory CA, Singh A. (2017). Household food security in the United States in 2016. USDA Economic Research Service. Retrieved from: <https://www.ers.usda.gov/webdocs/publications/84973/err-237.pdf>.
